# Cancer-specific and overall survival in patients with recurrent prostate cancer who underwent salvage extended pelvic lymph node dissection

**DOI:** 10.1186/s12894-016-0173-3

**Published:** 2016-09-06

**Authors:** Daniar K. Osmonov, Alexey V. Aksenov, David Trick, Carsten M. Naumann, Moritz F. Hamann, Amr Abou Faddan, Klaus-Peter Jünemann

**Affiliations:** Department of Urology and Pediatric Urology, University Hospital Schleswig-Holstein, Campus Kiel, Arnold-Heller Strasse 3, Haus 18, D-24105 Kiel, Germany

**Keywords:** Recurrent prostate cancer, Biochemical relapse, Salvage therapy, Lymph node dissection, Cancer-specific survival

## Abstract

**Background:**

The aim was to evaluate cancer-specific survival (CSS) and overall survival (OS) in patients with prostate cancer (PCa) recurrence who underwent salvage extended pelvic lymph node dissection (ePLND), taking into consideration pre- and postoperative androgen deprivation therapy (ADT).

**Methods:**

Salvage ePLND was performed in a cohort of 54 patients with PCa recurrence, and data from 45 patients were analyzed. The indications for salvage ePLND were biochemical recurrence (BCR) of PCa and suspect findings on ^11^C-choline PET/CT. PSA-level, biochemical response (BR), duration of biochemical recurrence freedom (BCRF), number of metastases, OS and CSS were analyzed retrospectively.

**Results:**

The average follow-up was 42.7 ± 20.8 months. Thirty-three patients (73.3 %, 95 % CI: 60.5–83.6 %) achieved BCRF during follow-up. The mean BCRF-period was 31.4 ± 19.7 months. CSS and OS were both 91.7 % ± 4.8 % (3-year survival) and 80.6 ± 8.6 % (5-year survival). Twenty-four patients (53.3 %, 95 % CI: 40.0–66.3 %) with castration-resistant PCa (CRPC) responded again to ADT after salvage ePLND.

**Conclusions:**

Salvage ePLND for selected patients with BCR and clinically recurrent nodal disease can achieve an immediate complete PSA response (i. e. BCRF) in nearly half of the patients. Patients with CRPC responded again to ADT after ePLND. Multicenter prospective studies with a control group are needed.

## Background

The standard treatment options for patients with prostate cancer (PCa) recurrence after radical prostatectomy (RP) are radiotherapy (RT) and/or androgen deprivation therapy (ADT) [1, 2]. Salvage extended pelvic lymph node dissection (ePLND) is neither mentioned in the European Association of Urology (EAU) guidelines, nor in the German S-3 and the US guidelines. However, it might be an alternative treatment approach in a selected group of the patients. Moreover, our data show that patients who had ceased to respond to ADT (i. e. CRPC patients) responded again to ADT after salvage ePLND.

RT is the most common option in cases of pelvic PCa recurrence. Salvage RT should be offered to patients with biochemical recurrence (BCR) or local recurrence after RP if there is no evidence of distant metastatic disease [3]. Briganti et al. performed a multicenter retrospective analysis of 472 node-negative patients who experienced BCR after RP and received salvage RT. The rate of 5-year BCR-free survival after early salvage RT was 73.4 %. The authors have developed the first nomogram to predict the outcome after salvage RT [4].

ADT is a standard method in node-positive patients with BCR after primary RP and RT [5]. Intermittent androgen deprivation results in non-inferior oncological efficacy when compared with continuous ADT in well-selected populations [2].

Sciarra et al. showed that 29.7 % of the patients with BCR after RP who received intermittent ADT developed castration-resistant prostate cancer (CRPC) and 14.2 % of the cases showed clinical progression, with a mean duration of 88.4 ± 14.3 months and 106.5 ± 20.6 months, respectively [6]. Prolonged ADT exposure increases the risk of cardiovascular disease and diabetes in men aged >75 years with PCa [7].

New substances such as abiraterone and enzalutamide represent new therapeutic options. Their mechanism of action regarding renewed ADT response is not clear, especially in consideration of insufficient control possibilities. In one-third of patients receiving abiraterone, the PSA level showed no tendency to decrease [8].

An alternative treatment option for patients with recurrent PCa, especially in CRPC, is salvage ePLND. The effect of the salvage surgery on the response to ADT after surgery is not clear. In the past 5 years there has been an increase in publications on the outcomes of salvage PLND. There is a partial or even a complete PSA response to salvage LND in patients with nodal recurrence of PCa. Despite the relatively small patient numbers and the lack of a long-term follow-up, the available data mark a preliminary success and demonstrate this technique to be a promising new approach [9–11].

The data presented in these and other similar studies were collected and analyzed in different centers and published independently of each other, but the outcome of all these studies was surprisingly similar. Salvage ePLND can take a more significant place in the treatment of patients with BCR. Some authors point out that a more extended type of PLND at the time of primary RP is to be favored. A high percentage of patients with pelvic LN recurrence responded with reduced PSA after salvage surgery, and thus, they might have already benefited from more extensive PLND during primary surgery [9, 10, 12].

In this single center retrospective study, we analyzed CSS and overall survival (OS) data, as well as the influence of salvage surgery on the response to ADT in patients with previous CRPC. This evaluation provides us with sufficient preliminary evidence to initiate a further study or to join a similar study with prospective randomized design.

## Methods

We analyzed a cohort of 54 patients with PCa recurrence. Most of the patients visited our clinic on their own initiative. They had expressed a wish to be treated in an alternative surgical way. All patients were informed about the absence of prospective multicenter data and about the fact that salvage ePLND is not mentioned in US, European or S3 guidelines.

All patients gave signed informed consent. Thirteen patients [28.9 %, 95 % confidence interval (CI): 18.2–41.9 %] were aged >70 years at the time of salvage ePLND. Recurrence was defined as an increase of PSA >0.5 ng/ml. We introduced salvage ePLND in Kiel in November 2003. We could eventually only evaluate the data from 45 of 54 patients, as the other patients continued follow-up treatment at their local urologist and we were unable to collect their data. The mean follow-up period was 42.7 ± 20.8 months.

Preoperatively, all patients underwent positron emission tomography (PET)/computed tomography (CT) imaging. ADT was aborted at least 4 weeks prior to PET/CT examination. There was no bone metastasis prior to salvage ePLND. The indications for salvage ePLND were BCR and/or positive PET/CT scan. Salvage ePLND was done in thirteen patients (37.1 %), who had undergone primary RP without LND; some of these had a negative PET/CT scan result, but all had a proven BCR.

We used the D’Amico risk classification to describe a primary tumor (Table 1). We defined an initial PSA level (iPSA) as a PSA level prior to primary treatment. Ten of 45 patients (22.2 %) had RT as primary treatment and underwent RP during salvage ePLND as well. Thirteen patients (28.9 %) had primary RP without any LND. In 22 patients (48.9 %) with primary LND, the mean number of removed LNs was 13.3 (range: 3–26) and LN metastases were found in five of those patients. R1 stage after primary prostatectomy were diagnosed in 12 (34.3 %) out of 35 patients. Gleason score (GS) subgroups were as follows: four (8.9 %) patients had GS 6; 17 (37.8 %) had GS 7; 10 (22.2 %) had GS 8; 10 (22.2 %) had GS 9; and two (4.4 %) had GS 10; and two (4.4 %) patients had a non-detectable GS (Table 1). Most patients had undergone primary RP ex domo.

**Table 1:** Summary of results of initial treatment (*n* = 35 with initial RP and *n* = 10 with initial RT, summary *n* = 45)

Total number of evaluated patients	45	100 %
Initial RT (%)	10	22.2 %
Initial RP without LND (%)	13	28.9 %
Initial RP with LND (%)	22	48.9 %
Average number of removed LN´s (*n* = 22)	13.3	3-26
Average number of N1 (%)	5	22.7 %
Mean iPSA, ng/ml (SD)	6.7	±1.6
Risk classification^a^		
Low (%)	1	2.2 %
Intermediate (%)	21	46.7 %
High (%)	21	46.7 %
T stage after RP		
T2 (%)	15	33.3 %
T3 (%)	27	60.0 %
T3a (%)	12	26.7 %
T3b (%)	15	33.3 %
Tx (%)	3	6.7 %
Gleason Score after RPGS 6 (%)		
GS 7 (%)	4	8.9 %
GS 8 (%)	17	37.8 %
GS 9 (%)	10	22.2 %
GS 10 (%)	10	22.2 %
GSx (non detectable) (%)	2	4.4 %
	2	4.4 %
R1 after initial PR (out of 35 patients) (%)	12	34.3 %

^a^In two patients, the initial risk group was unknown

We performed salvage ePLND according to the surgical template developed at our department [13]. The surgical regions of the Kiel template are defined as follows: (1) para-aortic LNs; (2) LNs along the common iliac artery; (3) LNs along the external iliac artery; (4) LNs along the internal iliac artery; (5) LNs in the Marcille’s triangle; (6) obturator LNs; and (7) presacral LNs. The Kiel principles of salvage ePLND are: (1) exclusively transperitoneal access; (2) definition of landmarks such as the iliac vessels prior to LND; and (3) careful separation of the ureter from the surrounding tissue. Subsequent to these measures, LND is systematically performed from the top downwards. Small or medium clips are used to avoid extensive ligation. Moreover, we used a harmonic scalpel to seal the lymph vessels and shorten the operation time [14]. The operation was performed no later than 4 months after PET/CT. None of the patients had bone metastases at the time of the intervention. The removed LNs were cut into 4-mm slices and examined further at our Pathology Institute.

PSA measurements were performed 40 days after surgery and thereafter every 3–6 months. In case of biochemical progression, PET/CT and bone scintigraphy were performed to exclude clinical progression. The data on the occurrence of bone metastases have been presented in our paper. There was no standard protocol for postoperative imaging diagnostics.

BR was defined as PSA regression immediately after salvage surgery, measured 40 days after surgery, regardless of whether the level was above or under BCR criteria. Some patients received ADT directly after salvage ePLND, prescribed by the local urologist. The patients who were followed up in our department received ADT on occurrence of renewed BCR after salvage ePLND. These patients have continuously received complete androgen blockage.

We analyzed the number of removed LNs, number of positive LNs, changes in PSA level, CSS and OS. Key points in the clinical outcome of salvage ePLND were BCR-free survival and its duration, as well as CSS and OS, taking into consideration the administration of ADT and the occurrence of bone metastases during follow-up. On analyzing the data, we found out that 80 % of the patients who underwent salvage ePLND had shown castration resistance (CRPC) prior to surgery, although CRPC was not a primary indication criterion for salvage surgery. We analysed the influence (whether negative or positive) of salvage ePLND + ADT on PSA recurrence after salvage ePLND in these particular patients.

95 % CIs for frequencies were determined based on the binomial distribution, with *p* = 0.05. Statistically significant differences of frequencies were determined by the exact variant of criterion χ^2^. Risk factors affecting survival were determined using Cox regression. CSS and OS were determined using Kaplan-Meier analysis.

The research was performed with the approval of the appropriate ethics committee (Ethics Commission, Faculty of Medicine, CAU Kiel University, reference number – D474/15).

## Results

**Fig. 1:**
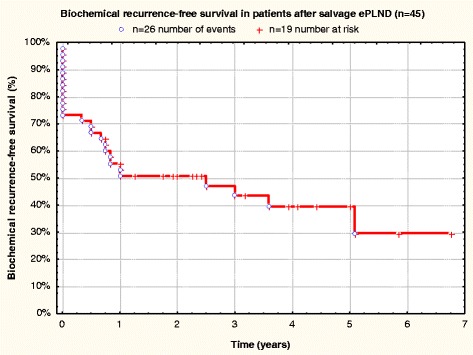
Biochemical recurrence-free survival in patients after salvage ePLND (*n* = 45)

**Fig. 2:**
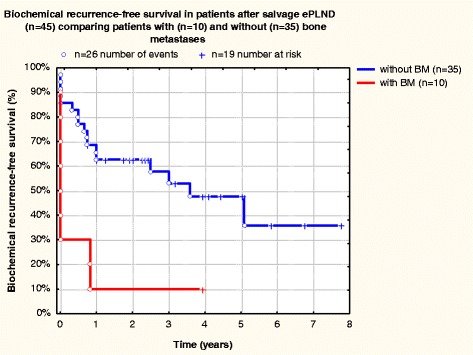
Biochemical recurrence-free survival in patients after salvage ePLND (*n* = 45) comparing patients with (*n* = 10) and without (*n* = 35) bone metastases

**Fig. 3:**
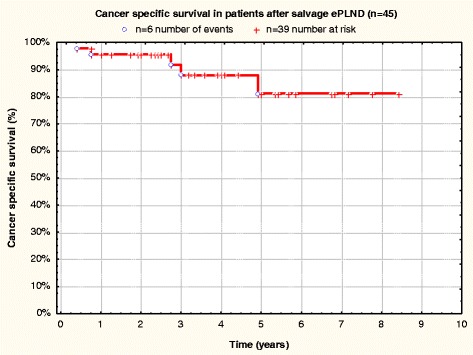
Cancer specific survival in patients after salvage ePLND (*n* = 45)

**Fig. 4:**
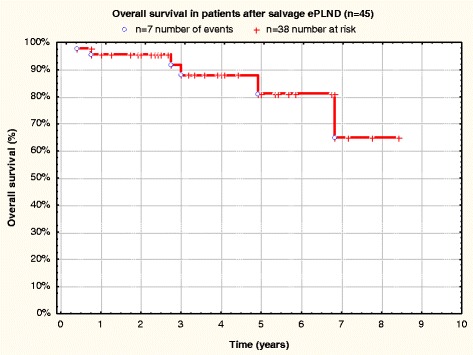
Overall survival in patients after salvage ePLND (*n* = 45)

The patient data are listed in Table 2 and Figs. 1, 2, 3 and 4. The PSA was measured ca. 40 days after salvage ePLND. At this point, 22 patients (48.9 %, 95 % CI: 35.8–62.1 %) were BCR-free. 16 of 22 patients (72.7 %) with immediate complete BR after salvage ePLND were treated with ADT prior to surgery. During follow-up, 33 patients (73.3 %, 95 % CI: 60.5–83.6 %) achieved BCR-freedom (BCRF), including those who received and responded to ADT again (Fig. 1) The mean follow-up in these 33 patients is 46.7 ± 25.0 months (median follow-up 47 months). In 10 patients (22.2 %, 95 % CI: 12.9–34.6 %), bone metastases appeared during follow-up. The mean duration until diagnosis of bone metastases was 25.9 ± 18.4 (median 21.5 months). Seven patients (15.6 %, 95 % CI: 8.0–26.8 %) died and six of these had bone metastases. In these patients, the average follow-up between salvage ePLND and occurrence of bone metastases was 34.2 ± 9.5 months [in this and similar cases, the data are represented as mean ± standard deviation (SD)]. BCR-free survival in patients with and without bone metastases was dramatically different (Fig. 2).

**Table 2:** Summary of results of salvage treatment (*n* = 45)

Follow-up (months), mean (SD)	42.7	(±20.8)
Median PSA-nadir after salvage ePLND, ng/ml (SD)	4.4	± 1.5
N-stage after salvage ePLND		
N0 (%)	19	(42.2 %)
N1 (%)	26	(58.7 %)
No. of LNs removed (range)	971	(4–76)
Mean no. of LNs removed per patient (SD)	21.6	(±9)
No. of positive LNs (range)	183	(1–42)
No. of positive LNs per patient (range)	4,1	(1–42)
No. of deaths (%)	7	(15.6 %)
Time to death (months), mean (range)	48.8	5–105
BR after salvage ePLND n (% patients)	31	(68.9 %)
BCRF immediately after salvage ePLND (complete BR) n (% patients)	22	(48.9 %)
BCRF during follow-up n (% patients)	33	(73.3 %)
Mean BCRF duration (months) (SD)	31.4	(±19.7)
ADT before salvage ePLND No. (%) patients	36	(80.0 %)
ADT after salvage ePLND No. (%) patients	40	(88.9 %)
Bone metastases in follow-up after salvage ePLND No. (%) patients	10	(22.2 %)

It is notable that the mean iPSA in patients without bone metastases was 5.5 ng/ml compared with 29.1 ng/ml in those with postoperatively diagnosed bone metastases. The mean PSA nadir after salvage ePLND was 4.7 ng/ml. We used an interval of 40 days for PSA nadir.

CSS and OS were equal at 91.7 ± 4.8 % (3-year CSS and OS) and 80.6 ± 8.6 % (5-year CSS and OS) (Figs. 3 and 4).

A total of 971 LNs were removed during 45 salvage ePLNDs, with a mean of 21.6 LNs (median 20, SD 9) per operation. Metastases were found in 183 (18.8 %) of 971 removed LNs. The mean number of positive LNs per patient was 4.1 (Table 2). In 22 patients (48.9 %, 95 % CI: 35.8–62.1 %) >20 LNs were removed per operation. In 26 patients (57.8 %, 95 % CI: 44.3–70.4 %), N1 stage was histologically confirmed. In four patients (8.9 %, 95 % CI: 3.7–18.3 %), just one LN was positive; in two patients (4.4 %, 95 % CI: 1.4–11.8 %) two LNs were positive; in 12 patients (26.7 %, 95 % CI: 16.4–39.5 %) 3–5 LNs were positive; in three patients (6.7 %, 95 % CI: 2.5–15.1 %) 6–10 LNs were positive; and in five patients (11.1 %, 95 % CI: 5.1–21.2 %) >10 LNs were positive (Table 3).

**Table 3:** Number of positive LNs per patient

No. of patients	Positive LNs per patient
19 (42.2 %)	Node-negative (N0)
4 (8.9 %)	1 LN+
2 (4.4 %)	2 LNs+
12 (26.7 %)	3-5 LNs+
3 (6.7 %)	6-10 LNs+
5 (11.1 %)	>10 LNs+
Summary: 45 patients	Mean LN+ pro patient 4.1 (range 1–42)

We have divided the patients into subgroups differentiating between pre- and postoperative ADT receivers and non-receivers and their post-operative ADT-response in Table 4.

**Table 4:** BCRF-survival in subgroups of patients according to pre- and postoperative ADT receivers and non-receivers and their postoperative ADT-response

		*Without* ADT after surgery	*With* ADT after surgery
Patients *without* preoperative ADT, *n* = 9 (20 %)	Total *without / with* ADT	3 (33.3 %)	6 (66.7 %)
	*Of the above*: BCRF-survival after salvage ePLND, n (%)	2 of 3 (66.7 %)	5 of 6 (83.3 %)
	Duration of BCRF-survival after salvage ePLND (months): 1. mean duration ± SD	18 ± 3	33.4 ± 15.8
	2. median duration	18	36
	3. min-max duration	15–21	6–53
Patients *with* preoperative ADT *n* = 36 (80 %)	Total *without / with* ADT	2 (5.6 %)	34 (94.4 %)
	*Of the above*: BCRF-survival after salvage ePLND, n (%)	2 of 2 (100 %)	24 of 34 (70.6 %)
	Duration of BCRF-survival after salvage ePLND (months): 1. mean duration ± SD	54.5 ± 5.5	30.1 ± 25.2
	2. median duration	51.75	23.5
	3. min-max duration	49–60	4–93

The following complications were observed in seven patients (15.6 %, 95 % CI: 8.0–26.8 %); all related to Grade IIIb Clavien–Dindo complications: two patients (4.4 %, 95 % CI: 1.4–11.8 %) with postoperative relevant bleeding: two (4.4 %, 95 % CI: 1.4–11.8 %) with ureteral stricture; one with rectovesical fistula (2.2 %, 95 % CI: 0.5–7.9 %), and two (4.4 %, 95 % CI: 1.4–11.8 %) with lymphocele.

We analyzed the risk factors for cancer-specific mortality. Only the occurrence of bone metastases during follow-up after salvage ePLND was shown to be a significant risk factor (Table 5).

**Table 5:** Risk factors of cancer-specific mortality (univariate analysis)

Risk factor (CSS)	p (significance)
Decrease of PSA level after salvage ePLND	0.268
Immediate BCRF after salvage ePLND	0.062
BCR	0.026
R-stage	0.253
T-stage	0.032
N-stage	0.299
Number of LNs	0.296
No. of LNs removed	0.42
Risk-group (D’Amico)	0.619
Bone metastases at follow-up	<0.001
Age at moment of salvage ePLND	0.249
Surgical complications	0.745

## Discussion

According to our results, we cannot postulate any benefit of salvage ePLND because of the limitation of the study, in particular its’ small retrospective character and heavily selected cohort of patients. Despite of that, the results have showed, that in the majority of patients BCRF were achieved, even in those with no histologically proven LN metastases. Moreover, CSS and OS data compared with acceptable complications rate makes this procedure feasible for selected patients. Similar results were found in previous studies on salvage ePLND. Thus, the data collected in different centers demonstrate comparable results [15] The most important two parameters are (1) complete biochemical response within 40 d after surgery (range: 46 %; 48.9 % up to 56.9 %) and (2) achievement of BCR-freedom after salvage ePLND with rates of up to 71.8 %. The role of ADT after salvage surgery remains a critical and unresolved issue, however.

The retrospective character of the study does not provide us with the opportunity to divide the patients into different groups with and without ADT. Moreover, the concept of recurrent PCa salvage treatment makes it impossible to split salvage ePLND and ADT; both treatments can and should be used in a combined approach. This approach has allowed us and other centers to achieve similarly high rates of CSS ranging between 75 % and 80.6 % [15].

We believe that the aim of any further research is to demonstrate that patients benefit from salvage ePLND. The selection of patients, taking into consideration the above-shown absence of reliable LN recurrence imaging tools, remains a matter of dispute.

13 (68.4 %) of 19 node-negative patients showed a BR immediately after salvage ePLND. 16 (84.2 %) reached BCRF during postoperative follow-up with a mean duration of 33 ± 22 months, median 28.5 (min 6 – max 81) months. However, 17 (89.5 %) undergone ADT after salvage ePLND in follow-up.

Again, 84.2 % patients with stage N0 after salvage surgery were BCR free. We discussed possible explanations for this with our pathologist and during various international meetings. For a better understanding, we also need to consider the method of pathological examination.

Pathological examination of removed LNs may miss micrometastases, which are defined in different ways depending on the cancer entity. In malignant melanoma, for example, one isolated tumor cell is already considered a micrometastasis according to the American Joint Committee on Cancer, although it can only be detected by means of immunohistochemistry. In breast cancer, micrometastases are defined as >200 μm (and/or >200 tumor cells) and <2 mm in diameter. Such micrometastases can be detected by light microscopy. In penile cancer, a micrometastasis is defined as being <2 mm [16].

There is no clear definition of micrometastasis with respect to PCa or cancer of other parenchymal organs. The International Society of Urological Pathology has no consensus on the definition of micrometastasis in PCa [17]. The problem is that something that is not clearly defined cannot be found.

However, we believe that we did remove LNs carrying micrometastases or even true metastases that were missed using the applied pathological method. We systematically removed all potentially positive LNs from various areas following the Kiel surgical template of salvage ePLND [14]. Most of the positive LNs were found in the area of the common iliac artery (37.5 %), paraaortal area (20 %), sacral area (12.5 %), and in the area of the triangle of Marcille (5 %). This is an important argument in favor of template-oriented surgery rather than selective or limited LN removal.

The small number of studies [15] shows the outcome of salvage ePLND, but it is more difficult to assess the difference in survival of patients after salvage ePLND alone versus patients after salvage ePLND and adjuvant ADT. In addition, it is difficult to assess the independent impact of these two therapeutic measures on progression-free survival. We observed an interesting phenomenon: in patients with CRPC and BCR, ADT can be effective again after salvage ePLND. We reported this side effect of salvage surgery at the 2014 meetings of the German Society of Urology, EAU and American Urological Association. This effect was detected after analysing all data and is a largely unexpected result of our study. We describe this effect with caution and as a preliminary statement. Moreover, we still lack an adequate explanation for this effect. However, we believe that this finding will have a major impact on the role of salvage ePLND if our data are confirmed by other study groups, ideally in a prospective setup with larger patient cohorts and in multicenter trials.

Our hypothesis so far is based on the tumor biology of PCa. The PCa tumor architecture is heterogeneous and probably depends on the different c-types and their mutation characteristics. There are a few theories about this heterogeneity, which can have an impact on the aggressiveness and speed of metastatic spread. In breast cancer, for example, the BRCA-1 and BRCA-2 genes indicate a strong dependency between BRCA-gene types and lymphatic and hematogeneous metastatic spread [18]. These two genes are found in LN or organ metastases of PCa. However, we believe that heterogeneity in PCa metastasis is even more complex. Based on the described facts, we see the potential reason for the renewed ADT response as the removal of aggressive tumor cells, which are probably chiefly responsible for metastatic spread of the tumor.

The interest in lesion-targeted salvage therapies has increased recently [19]. The outcome of salvage surgery and other diagnostic methods for positive LN detection compared to pathological examination needs to be evaluated further. Moreover, salvage surgery needs to be included in PCa guidelines. Lesion-targeted or selective salvage PLND can only be enhanced with development of new diagnostic imaging. Some recent studies have shown promising results with Ga-PSMA (^68^Gallium- Prostate-specific membrane antigen) PET/magnetic resonance imaging (MRI) and Ga-PSMA PET/CT [20, 21].

Most of the studies describing the clinical outcome of salvage ePLND are based on the findings of ^18^F choline PET/CT findings. Application of the tracer ^18^F-2-fluoro-2-deoxyglucose in PET/CT is successfully used in many tumor types. However, a benefit in PCa diagnosis has been questioned by several authors [22, 23].

There have been divergent results on choline-PET/CT regarding PCa recurrence. In several studies the detection rates were analyzed in relation to the PSA level. These studies have shown the sensitivity of choline-PET/CT for detection of LN metastases to be as low as 41.1 % [24]. Therefore, extensive salvage treatment is needed to maximize the chance of cure [19]. However, these results are insufficient to standardize the indications for salvage ePLND.

Data from our own department show a low specificity of 18.2 % with a relatively satisfactory sensitivity of 85.2 %. Most importantly, the positive predictive value and negative predictive value were 56.1 % and 50.0 %, respectively [25]. Therefore, we conclude that the reliability of PET/CT imaging for detection of LN metastases is limited by a high false-positive rate, and the findings in patients with low PSA values were associated with a high ratio of errors. In this situation, there is still no alternative to the template-based salvage ePLND to remove all LNs and thus to detect all metastatic LNs, including those that were false negative by PET/CT.

Here, as well as in the validation of the method mentioned above, a prospective study design should be set up for a full analysis of false-positive and false-negative results regarding LN metastasis. This will only be possible if histological verification is done after template-based ePLND.

Looking at the largest studies in this field, the CSS results in patients who underwent salvage ePLND seem to be similar despite the different cohorts analyzed by different workgroups. Thus, Jilg et al. reported 5-years CSS of 77.7 %, Rigatti et.al – 75 %, and our current data show 80.6 % [9, 10].

In the absence of evidence-based guidelines regarding salvage ePLND, our study and the publications listed in Table 1 allow us to conclude that salvage ePLND is effective and useful in selected patients. The only statistically significant finding in this study is that bone metastasis is associated with more rapid mortality. This is an important point which can help us define the indication criteria for salvage ePLND, but it should not be used to question the clinical significance of salvage surgery.

The requirements placed on the surgeon are high, however. The current situation remains unsatisfactory due to the lack of prospective multicenter randomized studies. We concede that our study was only a retrospective analysis. However, there are two good reasons for publishing these data. First, we needed to evaluate our own patient data in order to decide whether we can go further with salvage ePLND as a treatment option. Second, this analysis will serve as a basis for a prospective trial, for example, by joining in the SALPRO study of our colleagues from Freiburg, Germany. Finally, the low rate of complications that we found justifies further pursuit of this strategy.

## Conclusion

Salvage ePLND resulted in an immediate complete PSA response in nearly 50 % of the patients and in a BR in more than two-thirds. CSS and OS were equal at 91.7 ± 4.8 % (3-year CSS and OS) and 80.6 ± 8.6 % (5-year CSS and OS). In addition, one of the most important results of our study was the fact that 53.3 % of previous CRPC patients respond to ADT again after salvage ePLND. Bone metastasis is a poor prognostic surveillance factor after salvage ePLND. We do not wish to suggest that salvage surgery should be performed in patients with bone metastases, but we believe that salvage ePLND should find a place in the guidelines for selected groups of patients, especially as an additional option for CRPC patients. Multicenter prospective studies with control groups are needed to achieve a reliable output.

## Abbreviations

ADT: Androgen deprivation therapy
BCR: Biochemical recurrence
BCRF: Biochemical recurrence free(dom)
BR: Biochemical response
CRPC: Castration-resistant prostate cancer
CSS: Cancer specific survival
ePLND: Extended pelvic lymph node dissection
Ga-PSMA: ^68^Gallium- Prostate-specific membrane antigen
GS: Gleason score
iPSA: Initial PSA level
LHRH: Luteinising hormone-releasing hormone
LN(s): Lymph node(s)
LND: Lymph node dissection
OS: Overall survival
PCa: Prostate cancer
RP: Radical prostatectomy
RT: Radiotherapy
SD: Standard deviation
